# A Legal Decision

**Published:** 1890-07

**Authors:** 


### A LEGAL DECISION.

IN the Scotch papers is a curious legal point. A local farmer imported two Irish cows "just at the drop of carving." He sold them to a Dundee cowkeeper, who agreed to send for them next morning and take them away. During the night one calved and choked herself in the endeavor to eat the placenta. The cowkeeper refused to pay for the cow, and the sheriff held that he was justified in the refusal, inasmuch as there had been an absence of a right and proper degree of care and attention to the cow. The case was reheard, and evidence was given that the cow calved in the field; the farmer's servant saw that she had calved safely, and did not think it necessary to interfere. The judge held that the man was right, and condemned the cowkeeper to pay for the cow on the ground "that parturition being a natural operation, the less interference there is at and after, the better it generally will be."

